# Melioidosis: The hazards of incomplete peer-review

**DOI:** 10.1371/journal.pntd.0007123

**Published:** 2019-03-14

**Authors:** Direk Limmathurotsakul, Frances Daily, Sotharith Bory, Gaetan Khim, W. Joost Wiersinga, Alfredo G. Torres, David A. B. Dance, Bart J. Currie

**Affiliations:** 1 Mahidol Oxford Tropical Medicine Research Unit and Department of Tropical Hygiene, Faculty of Tropical Medicine, Mahidol University, Bangkok, Thailand; 2 Centre for Tropical Medicine and Global Health, Nuffield Department of Medicine, University of Oxford, Oxford, United Kingdom; 3 Diagnostic Microbiology Development Program, Phnom Penh, Cambodia; 4 Infectious Diseases Unit, Calmette Hospital, Phnom Penh, Cambodia; 5 Department of Medicine, Division of Infectious Diseases, Academic Medical Center, Amsterdam, The Netherlands; 6 Centre for Experimental and Molecular Medicine, Academic Medical Center, Amsterdam, The Netherlands; 7 The Herman Barnett Distinguished Professor in Microbiology and Immunology, Assistant Dean of Faculty Affairs and Professional Development, OFAPD, University of Texas Medical Branch, Galveston, Texas, United States of America; 8 Lao-Oxford-Mahosot Hospital Wellcome Trust Research Unit, Vientiane, Lao People’s Democratic Republic; 9 Menzies School of Health Research, Charles Darwin University and Infectious Diseases Department, Royal Darwin Hospital and Northern Territory Medical Program, Darwin NT, Australia; University of Washington, UNITED STATES

Sir,

As researchers working on *Burkholderia pseudomallei*, we were interested to see the review by Perumal Samy and colleagues [1], which highlights the significant clinical impact and public health threat caused by melioidosis in the tropics. However, although the general content of the abstract is valid, unfortunately the review itself contains considerable misleading, incomplete, and inaccurate information. Whilst not wishing to appear unduly critical, we have become aware that the review is already causing clinicians and microbiologists confusion, which could have a dangerous impact on the care of melioidosis patients. This was reinforced during our second Cambodia National Melioidosis Workshop in October 2017, with several participants noting that they had read the open-access review and were following some of the (dangerously) incorrect information therein. We, therefore, felt obliged to write to highlight the issue, which we feel reflects a failure of the peer-review process and the authors’ inadequate interpretation of recent advances in the melioidosis field. We have outlined some of the most important inaccuracies below.

In many ways, the errors and inaccuracies in the section ‘Antibiotic resistance and susceptibility/treatment of melioidosis’ are the most worrying, because they may lead to inappropriate management of patients with melioidosis and possibly unnecessary deaths. We witnessed definite evidence of this risk during preparations for the meeting in Cambodia. This section has a number of inaccurate points.

For example, on page 12, the statement “*B*. *pseudomallei* often develops resistance to existing antibiotics” is not true because, although acquired resistance has been reported, it is very rare with modern treatment regimens, and due to the absence of person-to-person spread, it represents a dead end when it does occur [2]. “However, there is varying susceptibility to the various antibiotics, such as chloramphenicol, tetracyclines, trimethoprim-sulfamethoxazole, ureidopenicillins, cephalosporins, and clavulanic acid” is also untrue, because most isolates have a remarkably consistent susceptibility profile [2,3], and also, clavulanic acid is never used alone. “Third-generation antibiotics, including cephalosporins, are not clinically useful in treating melioidosis” is misleading, because ceftazidime is generally considered the agent of choice for intensive phase treatment [4,5]. The statement “a treatment that combines chloramphenicol, doxycycline, and trimethoprim-sulfamethoxazole better controls the bacterium compared with individual treatment” completely ignores the results of several randomized controlled trials that show that this combination is inferior to ceftazidime for intensive phase treatment [6] and has no benefit over co-trimoxazole alone during the eradication phase [7,8]. “Fluoroquinolones…may be beneficial for immediate therapy or prophylaxis” and "Fluoroquinolones are given for treatment of acute melioidosis” are misleading because there is currently no role for fluoroquinolones in either treatment or prophylaxis of melioidosis [4,5]. On page 14, the statements “immunotherapy can significantly enhance the efficiency of conventional antimicrobial treatment for melioidosis,” “lower doses of antibiotics are essential for the successful management and can also play an important role in eliminating remaining bacteria in a short-term treatment course,” and “doxycycline can be used to treat localized melioidosis” are not supported by any evidence from clinical trials in humans, nor is there any evidence of a “synergistic effect of aminoglycosides and β-lactams” against *B*. *pseudomallei*. “Oral treatment consists of chloramphenicol, trimethoprim-sulfamethoxazole, and doxycycline” is not true because the current recommended oral eradicative treatment is co-trimoxazole alone, because it was shown that the addition of chloramphenicol and doxycycline made no difference to recurrence rates [7,8]. We have only listed some of the most striking examples. It might have been helpful for the authors to have referred to the international consensus guidelines on treatment that were published in 2010 or a more recent comprehensive review of this topic [4, 5].

The information provided in the “laboratory diagnosis” section is also incomplete and inaccurate, and could lead to both false-negative and false-positive diagnoses of melioidosis globally. On page 11, “Wrinkling of colonies is key to differentiation between *B*. *cepacia* (an opportunistic environmental pathogen) and *B*. *pseudomallei*; thus, further confirmatory tests, such as polymerase chain reaction (PCR), may be needed” is misleading. Not all strains of *B*. *pseudomallei* are wrinkled on all media, and this should not be used for identification. Furthermore, whilst it is true that PCR may be useful to confirm the bacterial identification [9], PCR is not routinely available in clinical microbiology laboratories in most developing countries, where melioidosis is highly endemic, so readers could be confused and request PCR when it is neither necessary nor available. In practice, what is needed in tropical developing countries are simple and pragmatic ways of making an accurate and rapid diagnosis. *B*. *pseudomallei* can reliably be identified by multiple methods, including latex agglutination, biochemical tests, and commercial identification systems such as the API20NE and Vitek II [9]. More recently still, a simple screen involving resistance to gentamicin and colistin and susceptibility to amoxicillin-clavulanic acid has been shown to be highly specific in Vietnam [10]. Although misidentifications are reported in some cases, simple methods can still perform satisfactorily with adequate training for microbiologists and technicians [9]. Some critical points about diagnosis are not mentioned in the review. These include (1) *B*. *pseudomallei* is commonly misidentified as a culture contaminant and discarded without any attempt at identification, especially by laboratory staff unfamiliar with this organism; (2) gram-negative bacilli that are oxidase positive, gentamicin- and colistin-resistant, and susceptible to amoxicillin-clavulanic acid should be strongly suspected to be *B*. *pseudomallei*; and (3) multiple methods may be required to identify *B*. *pseudomallei* [9]. On page 11, “However, blood cultures have been confirmed negative just before death in the septicemic form of infection” is misleading because many patients die within 24 to 48 hours of admission, and blood specimens collected from such patients before death are usually culture positive for *B*. *pseudomallei* [11,12]. “Detection of bacteria-specific antibodies from a blood sample is another form of diagnosis. Serological tests revealing high-antibody titers are very useful in the presence of clinical diagnosis” is inaccurate. It is well established that the most widely used antibody-based diagnostic test for melioidosis (indirect hemagglutination test [IHA]) is neither sensitive nor specific for diagnosis of melioidosis in endemic regions [13,14], and consensus guidelines for melioidosis diagnosis published in 2015 conclude that IHA is only likely to be useful in those from nonendemic regions who develop febrile illness following travel to a melioidosis-endemic area [9].

The review of global epidemiology is incomplete, unsystematic, and anecdotal and gives an underestimate of the potential burden of melioidosis in humans worldwide. Even though the review was published in May 2017, a number of key manuscripts published up to 2016 were not included or mentioned [15–20]. The methodology and criteria used for the literature review are not noted [1]. Table 1 “Global distribution of melioidosis” omits Lao PDR [16,17,21], where melioidosis is highly endemic, and also misses out Costa Rica, Gabon [21], Gambia, Kenya, Malawi, and Nigeria, where there have been sporadic case reports [15]. The numbers of reported cases for each country in brackets are also inaccurate [15–18]. For example, the number of published cases in Cambodia is 243, based on one of the most recent systematic reviews [15] with another 173 paediatric cases reported in a single paper published in 2016 [22], whereas the number is shown as only 5 in the review by Perumal Samy and colleagues [1]. On Page 4, Cambodia is included as a part of Vietnam [1], which is incorrect.

On page 1, the sentence containing the word “infrequently” might lead to the impression that melioidosis is infrequent in humans. The sentence should say “but (melioidosis is) also increasingly recognised as a common infectious disease in humans in many countries in the tropics” [15,17,23]. On page 2, “melioidosis is mainly transmitted by inhalation” is inaccurate because the relative contributions of inoculation, ingestion, and inhalation as routes of infections are currently unknown [24]. Although it is suspected that many melioidosis cases in Taiwan and Singapore may mainly be acquired by inhalation [25,26], it is thought that the main route of infection in agricultural developing countries is probably percutaneous inoculation [24,27]. On page 2, “However, central nervous system involvement in melioidosis is rare” is inaccurate because primary and secondary neurological involvement is not uncommon in Australia [19]. In addition, the sentence “This bacterium is relatively narrow in its worldwide distribution to the temperate regions” is misleading, because the disease is largely confined to tropical and subtropical areas, within which it appears to be quite widely distributed [15]. On page 3, “There was a total of 112 deaths, representing a fatality rate of 16.2%, during the period of 1998–2007 in Singapore [28], which is much higher than other countries like Thailand [29]” should say “which is much lower than other countries like Thailand, where a total of 956 deaths were observed between 1997–2006 in a single province, Ubon Ratchathani, in northeast Thailand, representing a case fatality rate of 42.6%, [29].” On page 5, in Australia, “The annual incidence was 5.8 cases per 100,000, whereas a higher incidence of 25.5 cases per 100,000 was noteworthy among native Australians” is obsolete because the most recent estimate of the annual incidence rate of melioidosis in the Top End of the Northern Territory is 19.6 per 100,000, with a higher annual incidence rate of 260 cases per 100,000 amongst diabetics [19,30]. On page 5, in Taiwan, “However, such infections (bacteremic pneumonia) were diagnosed only in 15 patients” is inaccurate. Reference 63 shows that bacteremic pneumonia was found in 10 of 15 (67%) culture-confirmed melioidosis patients in Taiwan from 1996 to 2000. The sentence is also misleading in saying that the incidence (total number) of melioidosis patients with bacteremic pneumonia is low. The most recent review from Taiwan reported a total number of 322 melioidosis cases occurring between 2002 and 2011 [31], and it is likely that 214 cases had bacteremic pneumonia during the period (67% of 322). Also, “Melioidosis should be included in the reportable diseases, and its prevalence in Taiwan should be monitored, as comprehensive data are lacking” is inaccurate because Taiwan is one of a few countries where all culture-confirmed melioidosis cases are mandatorily reportable to the national Centers for Disease Control (CDC). In Taiwan, melioidosis has been listed in the surveillance system since 2000 [31], and the data have been continuously comprehensive [31–33]. On page 6, regarding Indonesia, the systematic review and new indigenous case reports of melioidosis published in 2015 [18] is not mentioned, nor is a recent report of the increasing recognition of melioidosis in Sri Lanka [20]. On page 6, “However, no human case of melioidosis has been reported in Madagascar so far” is inaccurate because two indigenous melioidosis cases in Madagascar were published in 2014 [34]. The relevance of the discussion of glanders in the United States on page 7 is unclear in the context of this review.

In the section “Future perspectives,” some recommendations could lead to difficulties in management of melioidosis in resource-limited settings. On page 17, “Standard precautions include wearing masks, gloves, and gowns to prevent infection, especially among healthcare workers attending to patients with melioidosis” are not standard practice in many melioidosis-endemic countries, such as Thailand and Cambodia, and may lead to unnecessary fear among healthcare workers looking after melioidosis patients. Universal precautions are needed [35], but masks, gloves, and gowns are not routinely needed for healthcare workers in the absence of interventions associated with blood or body fluids. This is because human-to-human transmission is rare [5]. “After exposure to the causative organism, combination treatment with co-trimoxazole and doxycycline is recommended.” is inconsistent with the consensus guidelines on accidental laboratory exposure to *B*. *pseudomallei* and *B*. *mallei* published in 2008 [36], in which co-trimoxazole alone is the agent of first choice in the event of high-risk exposures only, with doxycycline or amoxicillin-clavulanic acid recommended if the organism is resistant to co-trimoxazole or the patient is intolerant.

The sections “Virulence and Pathogenesis” and “Development of antibodies and vaccines for prevention of melioidosis” are superficial and lack details of recent advances in the field. There are also other several points worthy of note. The virulence section, as presented, may mislead the readers that the invasive properties of the organism are mainly mediated by the type 3 secretion system (T3SS) and the presence of exotoxins and endotoxins that do not play a major role in the pathogenic process. Outstanding works by several research groups in recent years have defined the molecular mechanisms used by *B*. *pseudomallei* to survive within a host and the specific contribution of virulence factors to the pathogenesis of melioidosis [37]. Some of the key factors that were overlooked include activation of pattern recognition receptors by *B*. *pseudomallei* in the host cytosol, resulting in evasion of host cell autophagy. The knowledge that the intracellular environment within the cytosol signals activation of the type 6 secretion system (T6SS-1), allowing the organism dissemination; together with the T3SS-3 required for escape from the phagosome. Furthermore, T6SS-1 is critical for multinucleated giant cell (MNGC) formation. Finally, the role of exotoxins as a major virulence factor has not been demonstrated during infection in any validated model of infection and the endotoxin LPS is not considered a major virulence factor, and it is a common component of vaccine candidates [38]. Of interest, the authors state that the LPS of *B*. *pseudomallei* signals via Toll-like receptor (TLR)-5. We are not aware of any literature suggesting this may be the case.

In conclusion, this paper contains information that is inaccurate and recommendations for therapy that are not supported by current published guidelines for melioidosis. We hope that these comments will be useful to readers of the review [1] and have attempted to supplement this with some of the most recent evidence and current consensus guidelines for melioidosis management globally.
